# Vaginal Microbiota and Cytokine Microenvironment in HPV Clearance/Persistence in Women Surgically Treated for Cervical Intraepithelial Neoplasia: An Observational Prospective Study

**DOI:** 10.3389/fcimb.2020.540900

**Published:** 2020-11-05

**Authors:** Elisabetta Caselli, Maria D’Accolti, Erica Santi, Irene Soffritti, Sara Conzadori, Sante Mazzacane, Pantaleo Greco, Carlo Contini, Gloria Bonaccorsi

**Affiliations:** ^1^ Section of Microbiology, Department of Chemical and Pharmaceutical Sciences and LTTA Center, University of Ferrara, Ferrara, Italy; ^2^ Section of Gynecology and Obstetrics, Department of Morphology, Surgery and Experimental Medicine, University of Ferrara, Ferrara, Italy; ^3^ CIAS Research Center, University of Ferrara, Ferrara, Italy; ^4^ Section of Infectious Diseases and Dermatology, Department of Medical Sciences, University of Ferrara, Ferrara, Italy

**Keywords:** human papillomavirus, cervical neoplasia, surgical intervention, vaginal microbiome, vaginal cytokine, HPV clearance, HPV persistence infection

## Abstract

High-risk human papillomaviruses (hrHPVs) are causally related to cervical intraepithelial neoplasia (CIN) and subsequent cervical cancer (CC). The vaginal microbiome has been suggested to play a role in the development of CC, but the effect of conservative surgical treatment on the microbiome and hrHPV elimination has not been elucidated. In this study, we aimed to characterize the vaginal microbiome and inflammatory chemokine profile in 85 women treated for CIN2-CIN3 lesions, before and after surgical CIN removal. The results showed, as expected, a high prevalence of dysbiotic microbiomes and vaginal pro-inflammatory cytokines in the CIN cohort, correlated with disease severity, at the basal level. By contrast, surgical CIN removal induced significant vaginal microbiome variations, and specific microbiome/cytokine profiles were associated with hrHPV clearance/persistence at 6-month follow-up. hrHPV-cleared patients, in fact, showed a specific increase of *L. crispatus* and decrease of dysbiosis and inflammatory cytokines compared to hrHPV-persistent patients. These data highlight the crosstalk between HPV and the local microbiome, and suggest that vaginal microbiome modulation might represent a novel approach to modifying the natural history of hrHPV-related CC.

Study registration n. ISRCTN34437150 (https://www.isrctn.com/ISRCTN34437150).

## Introduction

Cervical cancer (CC) is one of the most common cancer in women, with an estimated 570,000 new cases in 2018 representing 7.5% of all female cancer deaths, and approximately 85% of the estimated 311,000 deaths per year occurring in low-income countries (Ferlay et al., 2018). In Italy, over 3,000 new cases were registered in 2018, with almost 1,000 deaths, despite the presence of screening and vaccination programs (Ferlay et al., 2019).

Human papillomavirus (HPV) infection is common in the female genital tract, and infection by high-risk oncogenic HPV (hrHPV) types has been causally related to invasive CC and its precursor premalignant stages (cervical intraepithelial neoplasia, CIN) (Ferlay et al., 2018; Ferlay et al., 2019). The virus is spontaneously cleared in most infections, and the persistence of oncogenic hrHPV infection, occurring in about 10% of infected women, is associated with carcinogenesis (Ferlay et al., 2018; Ferlay et al., 2019). However, hrHPV infection is a necessary but not sufficient condition for CC development, as many factors including immunodeficiency, age, smoking, sexual promiscuity, and concomitant virus or bacterial infections have been associated with higher persistence rates and oncogenic risk (Wheeler, 2008; Vriend et al., 2015; Seraceni et al., 2016; Tamarelle et al., 2019). Recently, a possible role of the vaginal microbiome as a cofactor in CC development was suggested (Di Paola et al., 2017; Kyrgiou et al., 2017; Champer et al., 2018). Studies on the human microbiome have, in fact, shown that commensal microorganisms can be a major factor in both health and disease, and such a role has also been recognized in the female genital tract. Contrary to most sites in the human body, where different microbial communities are generally considered a health signature, in the vaginal environment, eubiosis healthy status is characterized by a low degree of diversity (Boskey et al., 2001; Ravel et al., 2011), and dominance of few species of *Lactobacillus* (Ravel et al., 2011; Kroon et al., 2018), which can prevent the colonization of exogenous pathogens by producing lactic acid, bacteriocins and reactive oxygen species (ROS). According to the dominant species, vaginal microbiomes have been classified into five different Community State Types (CSTs) (Ravel et al., 2011), with L*. crispatus*, *L. gasseri*, *L. iners*, and *L.* *jensenii*, respectively, dominating CST-I, CST-II, CST-III and CST-V. In all the *Lactobacillus*-dominated microbiomes, the pH is typically <4.5, which is well tolerated by *Lactobacillus* but inhibits several other types of bacteria. By contrast, CST-IV is characterized by depletion of *Lactobacillus* spp and a significantly higher pH and bacterial diversity, with prevalence of anaerobic species, including *Gardnerella*, *Prevotella*, *Peptostreptococcus* genera, and/or aerobic bacteria of *Enterobacteriacee* (Pybus and Onderdonk, 1999; Turovskiy et al., 2011; Reid, 2016; Di Paola et al., 2017), frequently associated with bacterial vaginosis (BV), which is the most common vaginal infection in women of reproductive age (De Seta et al., 2019). On the other hand, BV is associated with increased risk of acquiring sexually transmitted infections including HPV-associated ones (Gillet et al., 2011; King et al., 2011; Gillet et al., 2012; Guo et al., 2012; Gosmann et al., 2017). CST-IV is also frequently associated with aerobic vaginitis (AV), where *Lactobacillus* spp. are predominantly replaced by group B *Streptococci* (GBS), *Escherichia coli*, and *Staphylococcus aureus* (Jahic et al., 2013a; Jahic et al., 2013b; Vieira-Baptista et al., 2016).

A CST IV microbiome profile has emerged as a potential risk factor for CC onset and progression (Torcia, 2019). Some taxa have emerged as being particularly associated with increased oncogenic risk, including *Ureaplasma parvum*, *Atopobium vaginae*, *Prevotella*, *Gardnerella*, *Sneathia sanguinegens*, and *Fusobacteria* (Brotman et al., 2014; Drago et al., 2016). Few data are otherwise available on non-bacterial components of the vaginal microbiome (mycetes, protozoa), although some reports have observed their association with increased CC risk (Liang et al., 2019).

The hypothesis that chronic inflammation may promote carcinogenesis is supported by the increased inflammatory cytokine levels found in patients with CC or its precursor premalignant stages (CIN) (Mhatre et al., 2012). Toward this hypothesis, recent metabolomics studies provided evidence of a metabolite interaction between the host and the microbiome, correlating glycochenodeoxycholate/carnitine metabolism with non-*Lactobacillus* dominance and genital inflammation, and a positive correlation between adenosine/cytosine and *Lactobacillus* abundance (Ilhan et al., 2019).

In women with high-grade CIN, Loop Electrosurgical Excision Procedure (LEEP) is the treatment of choice, being associated with a significant decrease in the risk of persistence at 6 months. However, no information is available on the impact of surgical resection on vaginal microbiome and chemokine profile. The present study was thus aimed to investigate the vaginal microbiome (including non-bacterial microorganisms) and cytokine profile in a cohort of women undergoing LEEP treatment for CIN2 or CIN3 lesions.

## Materials and Methods

### Study Design and Study Population

An oriented observational, prospective, cohort study, approved by the local Ethics Committee (Comitato Etico Unico della Provincia di Ferrara, Azienda Ospedaliero-Universitaria, Protocol N. 170394) (Study registration n. ISRCTN34437150, https://www.isrctn.com/ISRCTN34437150), was performed following The Code of Ethics of the World Medical Association (Declaration of Helsinki). Written informed consent was obtained from all enrolled women. Eighty-five women attending the Center of Preventive Gynaecology of the University-Hospital of Ferrara were enrolled. The eligibility criteria were age 30–50 years, CIN2/CIN3 diagnosis, candidate for LEEP, availability at 6-month follow-up, and signing informed consent. The exclusion criteria were pregnancy, innate or acquired immunodeficiency, concomitant neoplastic diseases or chronic inflammatory diseases/infections including aerobic vaginitis and other sexually transmitted diseases, corticosteroid, immune therapy, and unavailability at 6-month follow-up. HPV status was determined by Cobas 4800 HPV Test (Roche Diagnostics, Monza, Italy), allowing detection of hrHPV-16, 18, 31, 33, 35, 39, 45, 51, 52, 56, 58, 59, 66, 68. Patients were followed according to the Regional Protocol for CC prevention (Carozzi et al., 2015) and LEEP by monopolar energy diathermic loop was used for lesion removal. The protocol includes PAP-test, colposcopy and hrHPV-test before LEEP and follow-up control at 6 months after lesion removal. The CIN classification was performed by the unique central Pathological Anatomy laboratory of the University Hospital of Ferrara, based on the protocol defined for the CC prevention of Emilia Romagna region (Soloman, 1989; Darragh et al., 2013; GISCI, 2018).

### Sample Collection

Cervico-vaginal samples were collected using sterile rayon swabs before LEEP treatment and during follow-up control, then put in 0.4 ml of sterile saline in a 1.5-ml sterile microtube, which was immediately refrigerated and processed within 3 h. Vaginal secretions were collected by washing the vaginal cavity with 5 ml of sterile saline. The collected samples were aliquoted in sterile 1.5-ml microtubes, immediately refrigerated, and then frozen at −80°C until use.

### DNA Analysis

The total DNA was extracted from the cervico-vaginal swab samples using the Exgene Cell SV Kit (Gene All, Tema Ricerca, Bologna, Italy), following the manufacturer’s instructions, preceded by a pre-lysis step with 5 mg/ml of lysozyme to obtain optimal lysis of Gram-positive bacteria, as previously described (Comar et al., 2019). The extracted DNA was quantified by spectrophotometric reading at 260/280 nm, using a nanodrop (Thermo Scientific, Milan, Italy). The quality and amplificability of extracted DNA was checked by polymerase chain reaction (PCR) amplification of the human beta-actin house-keeping gene (for eukaryotic DNA) and bacterial 16S rRNA gene (panbacterial PCR, *panB*, for prokaryotic DNA), as previously described (Caselli et al., 2018). The total DNA extracted from clinical samples was analyzed *via* a real-time quantitative PCR (qPCR) microarray targeting 90 species usually present in the lower female genital tract, including bacteria, mycetes and protozoa (Microbial Vaginal Flora Array, catalog n. BAID-1902ZRA-24; Qiagen, Hilden, Germany). An amount of 1 µg of extracted DNA per plate (10 ng/well/reaction) was used for each microarray analysis (corresponding to 10 ng per reaction per well). Negative controls were included in each microarray assay, consisting of the microarray NTC (No Template Control) (Qiagen, Hilden Germany). The relative quantitation of each individual microbial parameter was calculated by Qiagen software (https://geneglobe.qiagen.com/ca/analyze/) and expressed as Log_10_ fold change compared to the Ct values detected in the NTCs.

### Cytokine Analyses

Individual aliquots of vaginal washings were thawed on ice, and 50 µl of undiluted vaginal washings were first analyzed by a multiplex cytokine array (Multi-Analyte ELISArray, catalog n. MEH-004A; Qiagen, Hilden, Germany), following the manufacturer’s instructions. This ELISA array allowed us to simultaneously detect 12 different cytokines/chemokines: IL1α, IL1β, IL2, IL4, IL6, IL8, IL10, IL12, IL17α, IFNγ, and TNFα. Subsequently, based on the multiplex ELISA results, individual ELISA assays were performed for each cytokine/chemokine detected in the vaginal washing samples, namely, IL1α (BMS243-2, Hu IL-1A coated ELISA), IL1β (BMS224-2, Hu IL-1B coated ELISA), IL6 (KAC1261, Hu IL-6 ELISA kit), IL8 (KHC0081, Hu IL-8 ELISA kit), and TNFα (BMS2034, Hu TNFa coated ELISA) (all from Thermo Fisher Scientific, Monza, Italy). The assays were performed following the manufacturer’s instructions and using 50 µl of undiluted sample per well. All the samples were assayed in triplicate in the multiplex ELISA array and in duplicate in individual ELISA assays.

### Statistical Analyses

GraphPad Prism (v. 5) was used for statistical data analysis. Student’s *t* test and Mann-Whitney test were used for comparison of two groups, and ANOVA and Kruskal-Wallis tests for comparison between groups. Pearson’s chi-squared test was used for correlation analysis. A *p* value < 0.05 was considered significant.

## Results

### Study Cohort

The demographic and clinical characteristics of the study population of the CIN cohort enrolled in the study are summarized in **Table 1**. Eighty-five patients were enrolled, with an average age corresponding to 38.02 years (median value 38; range 30–50); 78 of these patients were Caucasian, thus representing the vast majority of the study population (94.8%). Smokers accounted for one third of the total study cohort. Respectively, patients diagnosed as CIN2 and CIN3 represented 48.2% and 51.8% of the whole group of enrolled patients. As expected, the majority of enrolled patients (72/85, 84.7%) were positive for hrHPV, as determined by routine testing, while 13 patients were hrHPV-negative.

**Table 1:** Characteristics of the CIN2/CIN3 patients enrolled in the study.

Demographic features	Study population (n, %)
Total n.	85
Age (mean, range)	38 (30–50)
BMI (mean, range)	22.8 (16.5–34)
Smoke (n, %)	28 (32.9%)
Ethnicity (n, %)	
*Caucasian*	78 (94.8%)
*African*	1 (1.2%)
*Asiatic*	3 (3.5%)
*Hispanic*	3 (3.5%)
Deliveries (n, %)	
*Nulliparous*	35 (41.2%)
*Parous*	49 (57.6%)
Contraceptives (n, %)	31 (36.5%)
*Oral*	22 (25.9%)
*Ring*	6 (7.1%)
*IUD*	3 (3.5%)
Pap test (n, %)	
*Negative cytology*	5 (5.9%)
*ASC-US*	2 (2.4%)
*ASC-H*	14 (16.5%)
*L-SIL*	40 (47.1%)
*H-SIL*	24 (28.2%)
Colposcopy (n, %)	
*NTZ*	6 (7.1%)
*anTZ-G1*	43 (50.6%)
*anTZ-G2*	36 (42.3%)
Histology (n, %)	
*CIN2*	41 (48.2%)
*CIN3*	44 (51.8%)
hrHPV-positive (n, %)	72 (84.7%)

BMI, Body Mass Index (kg/[m]^2^); IUD, Intra Uterine Device; ASC-US, Atypical Squamous Cells of Undetermined Significance; ASC-H, Atypical Squamous Cells, cannot exclude high-grade lesion; L-SIL, Low-grade Squamous Intraepithelial Lesion; H-SIL, High-grade Squamous Intraepithelial Lesion; NTZ, Normal Transformation Zone; anTZ-G1, abnormal Transformation Zone Grade 1; anTZ-G2, abnormal Transformation Zone Grade 2.

Given that the study aim was to assess the association between the vaginal microbiome and hrHPV persistence in CIN patients, the hrHPV-negative women were excluded from the subsequent analyses. Sixty-six patients completed the follow-up (66/72, 91.7%). Among the hrHPV-positive patients who completed the follow-up, 16 showed margin involvement at the basal level and three of them showed recurrent lesions at follow-up.

Each enrolled patient was subjected to two samplings, one at the enrollment, just before the surgical excision of the cervical lesion, and one 6 months later, during the follow-up phase. From each patient one vaginal swab and one vaginal washing were collected, and these were analyzed for their microbiome profile and cytokine pattern, respectively.

### Microbiome Characterization in the CIN Study Cohort

#### Microbiome Profile at the Basal Level

**Figure 1:**
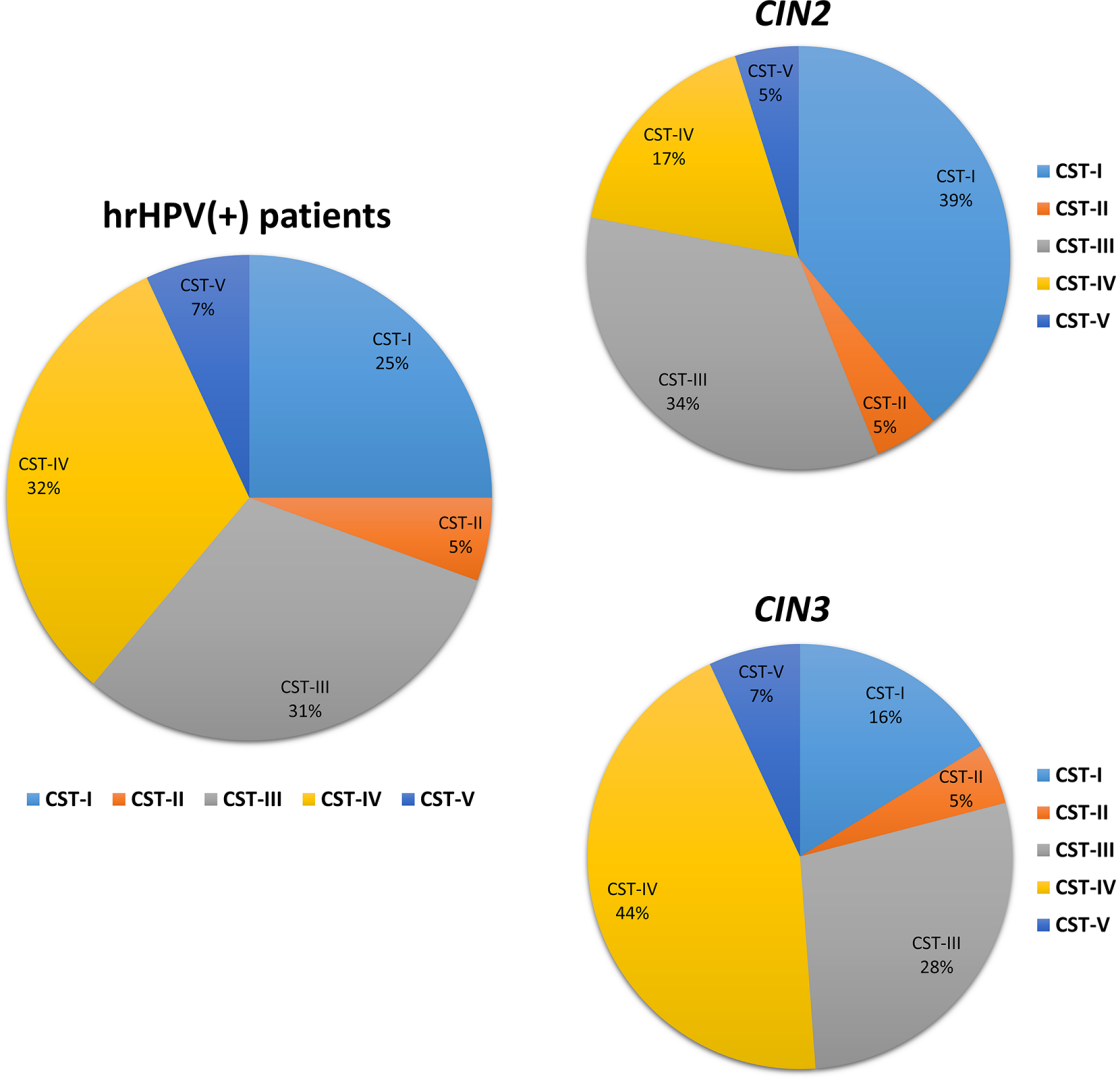
Distribution of the Community State Types (CSTs) in the CIN study cohort. Total DNA extracted from vaginal swabs was analyzed by a specific qPCR microarray simultaneously identifying and quantifying 90 microbial species of the vaginal tract. CSTs were defined according to the prevalence of Lactobacilli or other bacteria. The results are expressed as the percentage of each CST among all hrHPV-positive patients (total number = 72), CIN2 patients (n = 34), or CIN3 patients (n = 38).

The microbiome profile was analyzed in the total DNA extracted from vaginal swabs by using a real-time quantitative polymerase chain reaction (qPCR) microarray, identifying at the species level and simultaneously quantifying 90 microbial species usually present in the vaginal tract. Besides the bacteriome, this method also allowed the identification of mycetes and protozoa. The characterization of vaginal microbiomes by microarray allowed for recognition of the five vaginal CSTs within the study cohort (**Figure 1**). Overall, 18/72 (25.0%) of the vaginal microbiomes were identified as CST-I, 4/72 (5.5%) as CST-II, 22/72 (30.6%) as CST-III, 23/72 (32.0% %) as CST-IV, and 5/72 (6.9%) as CST-V. The CST distribution differed significantly in the CIN 2/CIN3 subgroups, with a statistically significant increase of CST-IV in CIN3 compared to CIN2 patients (17 vs. 44%, *p* < 0.001), and a concomitant decrease of CST-I (39 vs. 16%, p < 0.001).

**Figure 2:**
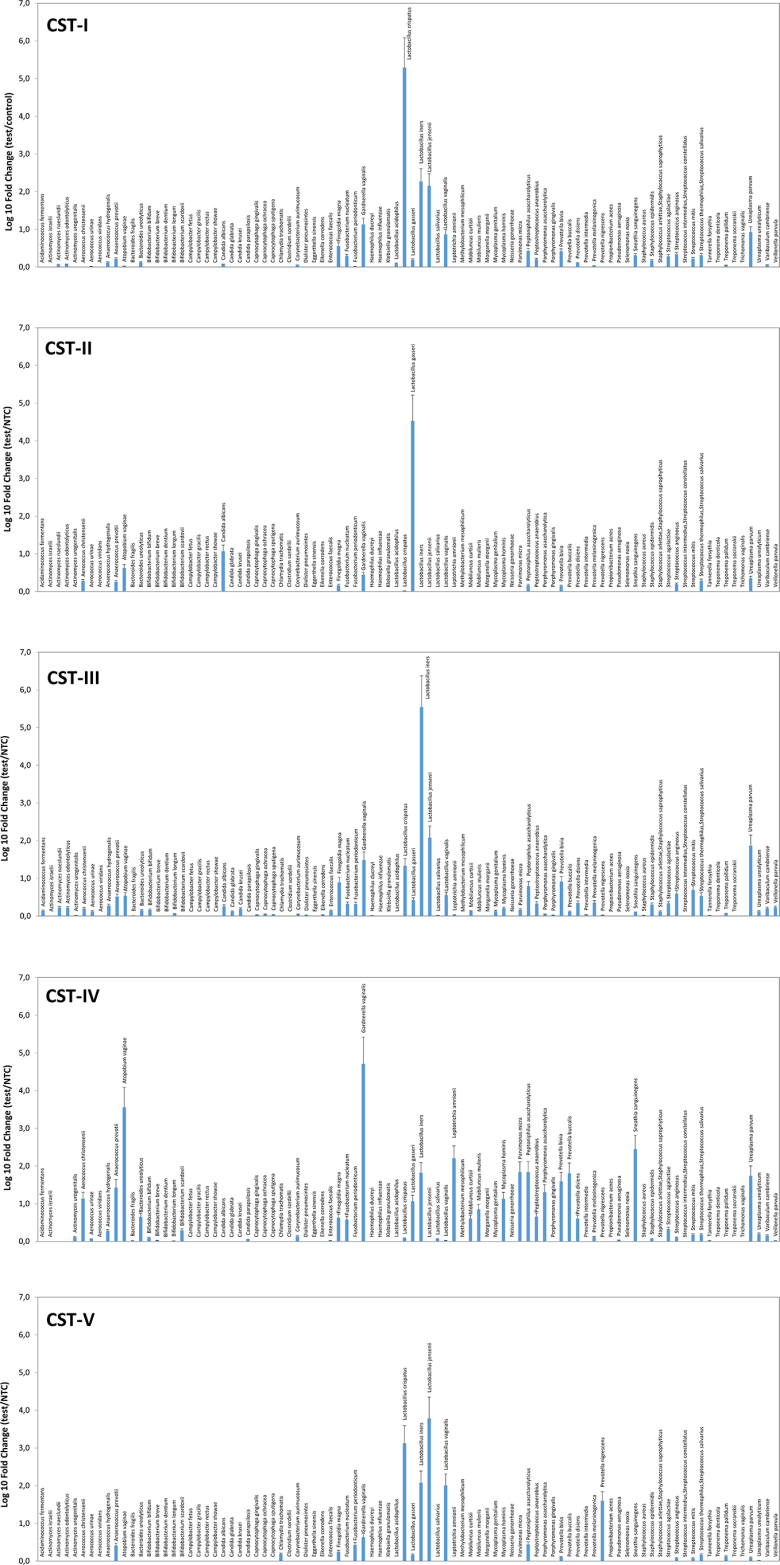
Microbiome profiles of the five CSTs in the CIN study cohort. The results were obtained by analyzing total DNA extracted from vaginal swabs by a specific qPCR microarray simultaneously identifying and quantifying 90 microbial species of the vaginal tract and are expressed as Log_10_ fold change compared to negative controls (NTC, no template controls). Mean values ± SD for each microbial parameter are shown.

Similarly, the species prevalence and amount differed significantly among the different CSTs (**Figure 2**). In particular, the CST-I cluster was characterised by, besides *L. crispatus* dominance, the simultaneous high prevalence of other Lactobacilli, and by detectable levels of *F. magna*, *G. vaginalis*, and *U. parvum* (5.84, 21.66, and 13.62 fold-change, respectively, compared to negative controls).

The CST-II group was instead characterised by the almost total absence of Lactobacilli, except for *L. gasseri* (dominant), and fair amounts of *A. vaginae* and *C. albicans* (respectively, 5.54 and 15.54 fold compared to controls). CST-III microbiomes contained, besides dominant *L. iners*, other less abundant Lactobacilli, and *U. parvum* was particularly abundant (68.45 fold, compared to negative controls). *F. magna*, *G. vaginalis*, *Peptoniphilus asaccharolyticus*, *Prevotella bivia*, and some *Streptococcus* species (*agalactiae*, *anginosus*, *mitis*, *thermophilus/salivarius*) were also present. The CST-IV cluster was characterised by a mixed population harbouring low or no Lactobacilli and high amounts of BV-associated microorganisms, including *Aerococcus christensenii* (13.01-fold), *Anaerococcus* *prevotii* (25.38-fold), *A. vaginae* (3428.12-folds), *Leptotrichia amnionii* (152.11-fold), *Mycoplasma hominis* (12.83-fold), *Parvimonas micra* (64.93-fold), *Peptoniphilus* *asaccharolyticus* (65.58-fold), *Porphyromonas* *asaccharolitica* (19.28-fold), *Prevotella bivia* (36.99-fold) and *buccalis* (60.64-fold), *Sneathia sanguinegens* (266.03-fold), and *U. parvum* (52.28-fold). *F. magna*, *F. nucleatum*, *Mobiluncus curtisii* and *mulieris*, and *Peptostreptococcus* *anaerobius* were also detectable (about 5-folds each, compared to negative controls). The CST-V group (*L. jensenii* dominant) showed fairly abundant *L. crispatus*, *iners*, and *vaginalis*, and elevated presence of *Prevotella nigrescens* above all, with very scarce amounts of the microorganisms detected in the other CST profiles. No protozoa were detected in any sample.

**Figure 3:**
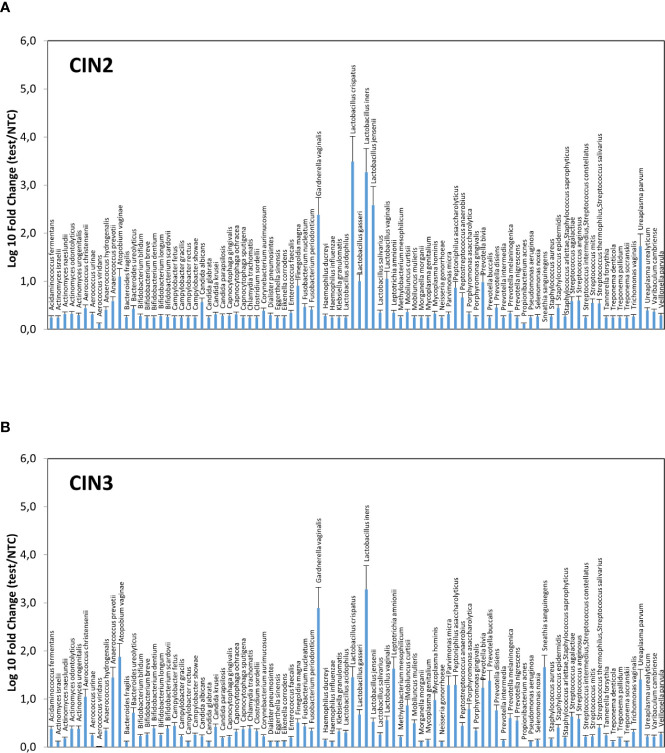
Microbiome profiles of the CIN2 **(A)** and CIN3 **(B)** subgroups. The results were obtained by analyzing the total DNA extracted from vaginal swabs by a specific qPCR microarray simultaneously identifying and quantifying 90 microbial species of the vaginal tract, and they are expressed as Log_10_ fold change compared to negative controls (NTC, no template controls). Mean values ± SD for each microbial parameter are shown.

Significant differences were observable in grouped CIN2 and CIN3 patients (**Figures 3A, B**), with the CIN2 subgroup having a microbiome still dominated by *Lactobacillus* spp., though with a high presence of anaerobic Gram-negative BV-associated bacteria (especially *A. vaginae*, *G. vaginalis*, and *U. parvum*) and less prevalent microbes, including *C. albicans*, *F. magna*, *Peptoniphilus asaccharoliticus*, *Peptostreptococcus anaerobius*, *Prevotella bivia*, and some Streptococci. By contrast, the CIN3 microbiomes showed a drop in Lactobacilli, except for *L. iners*, and high prevalence of *A. vaginae*, *G. vaginalis*, and *U. parvum*, accompanied by other species not frequently detectable in CIN2 patients, including *Aerococcus christensenii*, *Anaerococcus* *prevotii*, *Leptotrichia amnionii*, *Mycoplasma hominis*, *Parvimonas micra*, *Peptoniphilus* *asaccharolyticus*, *Porphyromonas* *asaccharolitica*, *Prevotella bivia* and *buccalis*, and *Sneathia sanguinegens* (this last species at 46-fold compared to controls).

**Figure 4:**
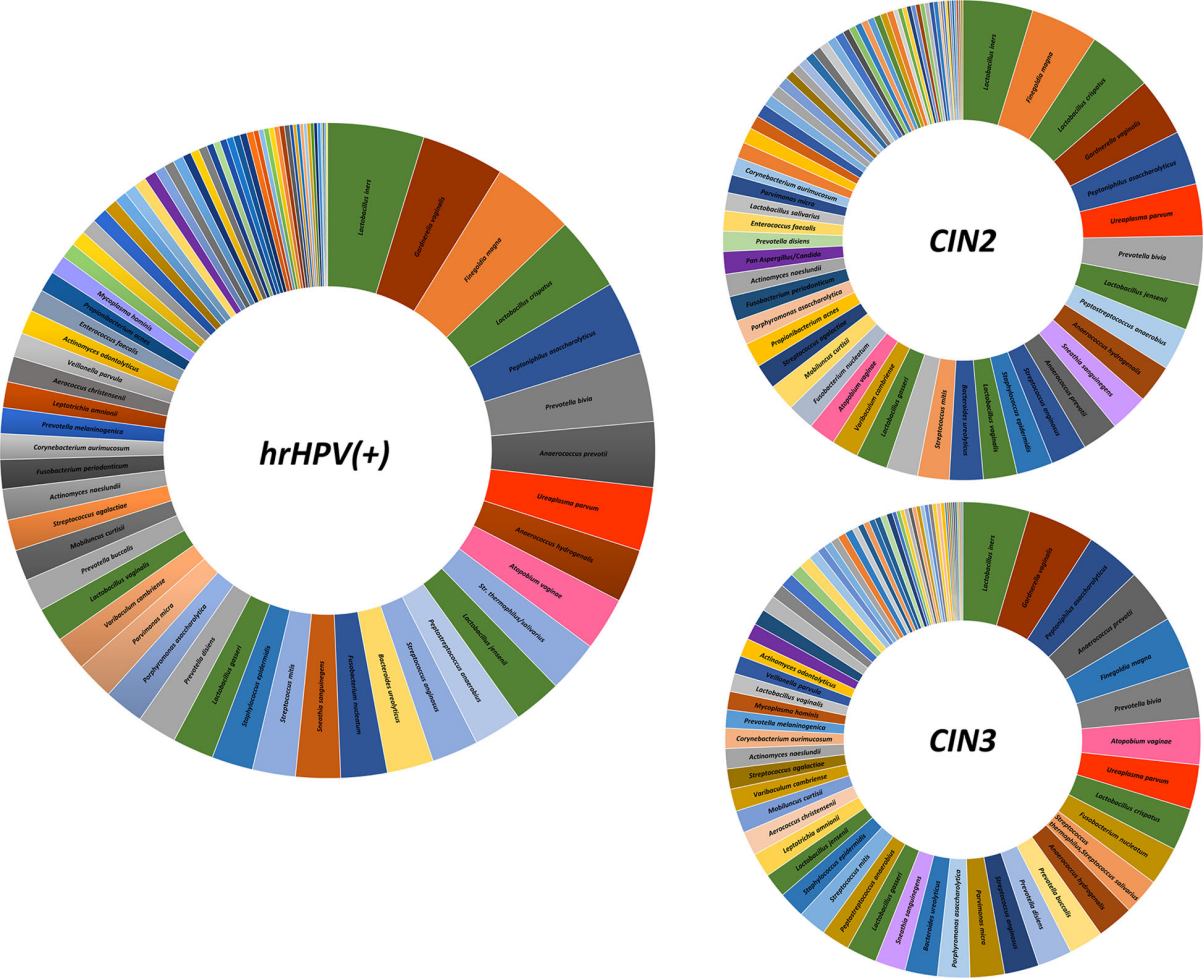
Frequency of detection of microbial species in the CIN study cohort. The results, obtained by qPCR microarray analysis of the vaginal microbiome, are expressed as percentages of detection frequency for the indicated species in the whole CIN study group (72 patients) and in the CIN2 and CIN3 subgroups (34 and 38 patients, respectively).

Overall, the most frequently detected species in CIN patients was *L. iners* (76.7% of samples), followed by *G. vaginalis*, *F. magna*, *L. crispatus*, *P. asaccharolyticus*, *P. bivia*, *A. prevotii* and *U. parvum*, present in > 50% of CIN samples (**Figure 4**). Compared to CIN2 patients, the CIN3 subgroup had significantly increased frequency of *G. vaginalis* (75% vs. 63%) and *A. vaginae* (52% vs. 29%), and decreased *F. magna* (59% vs. 73%). Furthermore, *Lactobacillus* spp. other than *L. iners* were much less frequent in CIN3 compared to the CIN2 group, with *L. crispatus* dropping from 70% in CIN2 to 47% in the CIN3 group, and *L. jensenii* from 48% in CIN2 to 27% in the CIN3 group. Also, *F. nucleatum*, *P. micra*, *P. disiens/buccalis*, *L. amnionii*, *M. hominis*, *U. urealyticum*, and *C. trachomatis* were increased in CIN3 compared to the CIN2 group, and, contrarily, *S. epidermidis*, *L. vaginalis* and *salivarius*, *P. acnes*, and *Candida krusei* were less frequent in CIN3 compared to the CIN2 samples.

To provide more detail, **Table S1** reports the percentage values detected in the whole hrHPV-positive study cohort, as well as in the CIN2 and CIN3 subgroups.

#### Microbiome Profile at Follow-Up

Most patients completed the 6-month follow-up after surgical intervention (66/72, 91.6%). Virus persistence was found in 14 patients (21.2%), and 4 of them had clinical CIN2/CIN3 recurrence (4/14, 28% recurrence rate); instead, 78.8% of the patients were hrHPV-negative, with a 0% clinical recurrence rate among them. All the 4 recurrence cases were hrHPV-positive at 6-month follow-up, and 3 of them had previous margin involvement.

**Figure 5:**
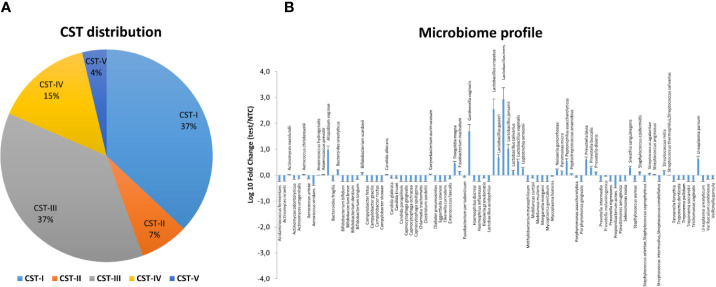
Vaginal microbiome profile of the CIN study group at follow-up. The total DNA extracted from vaginal swabs was analyzed by a specific qPCR microarray simultaneously identifying and quantifying 90 microbial species of the vaginal tract. **(A)** CST distribution in follow-up patients (66 patients). The results are expressed as the percentage of each CST among all patients who completed the follow-up. **(B)** Microbiome profile of the whole follow-up group of patients (66 patients). The results were obtained by qPCR microarray, and are expressed as Log10 fold change compared to negative controls (NTC, no template controls). Mean values ± SD for each microbial parameter are shown.

**Figure 6:**
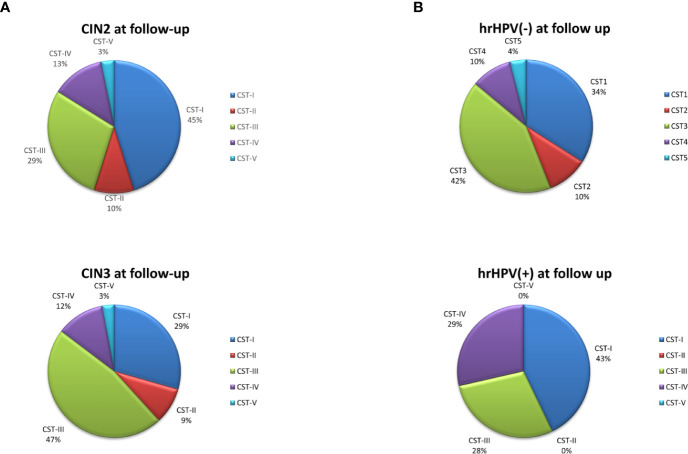
CST distribution in follow-up patients, as detected by qPCR microarray analysis. **(A)** Patients subdivided for lesion degree (CIN2 or CIN3) before intervention. The results are expressed as the percentage of each CST among all CIN2 (n = 34) and CIN3 (n = 38) patients. **(B)** Patients subdivided for hrHPV-status at follow-up. The results are expressed as the percentage of each CST among all hrHPV-negative (n = 52) or hrHPV-positive (n = 14) patients at follow-up.

The follow-up cohort showed significant modification of the CST distribution compared to the basal profiles (**Figure 5A**), with a significant increase of CST-I (37 vs. 25%, p < 0.001) and concomitant decrease of CST-IV (15 vs. 33%, *p* < 0.0001). CST-II and CST-V were unvaried, whereas CST-III was increased, although not significantly. Species analysis evidenced an overall increase of Lactobacilli, mostly *L. crispatus* (930-fold increase compared to basal levels), although *L. iners* was still the most prevalent. Concomitantly, several species detected at the basal level decreased, including *A.* *vaginae*, *U. parvum*, and *G. vaginalis* (**Figure 5B**). Interestingly, the CST distribution at follow-up differed both in CIN2 versus CIN3 subgroups (**Figure 6A**) and in hrHPV-cleared versus hrHPV-persistent patients (**Figure 6B**). In particular, CST-IV decrease compared to basal levels was more evident in CIN3 (from 44 to 12%, *p* < 0.001) compared to CIN2 patients (from 17 to 13%, *p* < 0.05), and am unmodified proportion of the hrHPV-persistent group presented the CST-IV profile (*p* = ns, not significant). The CST-I increase was higher in CIN3 (from 16 to 29%, *p* < 0.001) than in the CIN2 group (from 39 to 45%, *p* < 0.01), while the reverse was observed for CST-III, which was more prevalent in CIN3 compared to CIN2 patients (47 vs. 29%, *p* < 0.001). Concerning the hrHPV-status at follow-up, the results showed that a significantly decreased proportion of hrHPV-cleared patients had the CST-IV profile (from 32 to 10%, *p* < 0.0001), while the hrHPV-persistent group presented an unmodified prevalence of the CST-IV profile (32 vs. 29%, *p* = ns), although an increase of the CST-I type was also detectable in this subgroup. No significant differences in the proportion of virus clearance were observed relative to the basal CST groups, although the low number of patients in some CSTs rendered comparison between the populations difficult.

**Figure 7:**
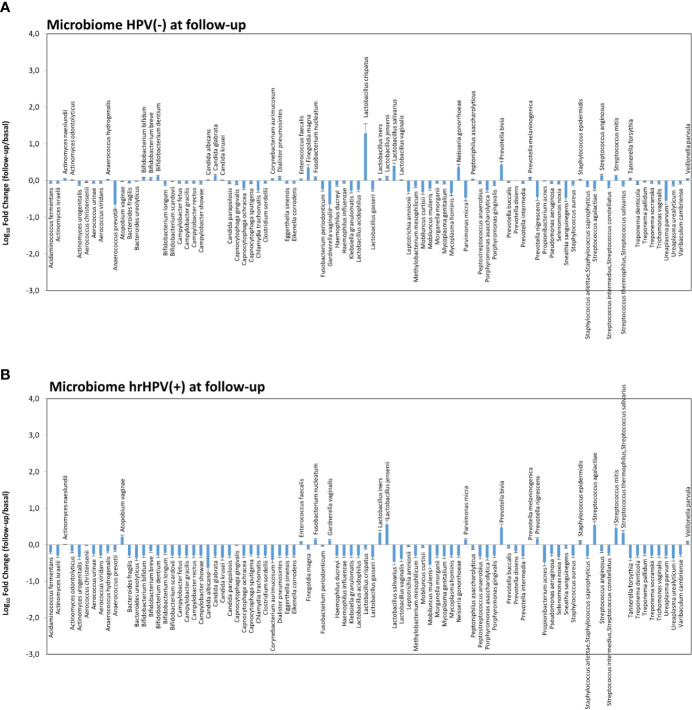
Microbiome profiles detected in hrHPV-negative **(A)** and positive **(B)** subgroups at follow-up. The results, obtained by qPCR microarray analysis of the vaginal microbiomes, are expressed as Log_10_ fold change compared to the corresponding basal levels detected before intervention in each subgroup. Mean values ± SD for each microbial parameter are shown.

Notably, the variations observed in the microbiome composition of the whole follow-up cohort were entirely ascribable to the hrHPV-cleared group, showing a significant increase of *L. crispatus* (19.3 fold compared to the basal amount, *p* < 0.001) and a concomitant decrease of BV-associated species, including *A. vaginae*, *G. vaginalis*, and *U. parvum* (**Figures 7A, B**), whereas little or no modifications were observed in the microbiome profiles of the hrHPV-persistent patients.

**Figure 8:**
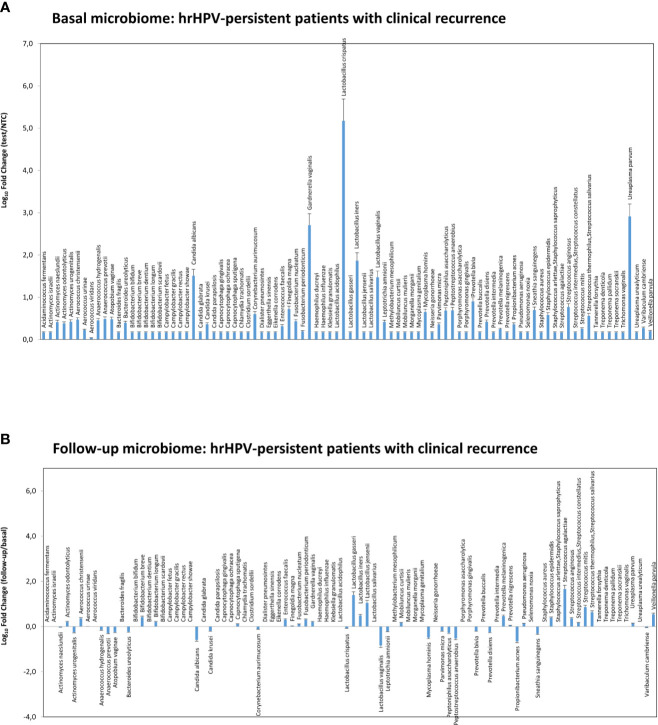
Microbiome profiles detected in the hrHPV-persistent patients with clinical CIN2/CIN3 recurrence at follow-up. The results were obtained by qPCR microarray analysis of vaginal microbiomes. **(A)** The basal microbiome results are expressed as Log_10_ fold change compared to negative controls (NTC, no template controls). **(B)** The follow-up microbiome results are expressed as Log_10_ fold change compared to the values detected at the basal level. Mean values ± SD for each microbial parameter are shown.

At 6-month follow-up after LEEP, four patients with persistent HPV infection showed CIN2 or CIN3 recurrence and required further surgical treatment. Those recurrent patients exhibited a CST-I profile at the basal level (*L. crispatus* dominant), yet were characterised by very high loads of *G. vaginalis*, *U. parvum*, and *C. albicans*, compared to the whole CST-I group, and no significant variations were detected at follow-up, except for a slight decrease of *L. crispatus* and increase of *S. agalactiae* (**Figures 8A, B**).

### Vaginal Cytokine Profile in the CIN Study Cohort

**Figure 9:**
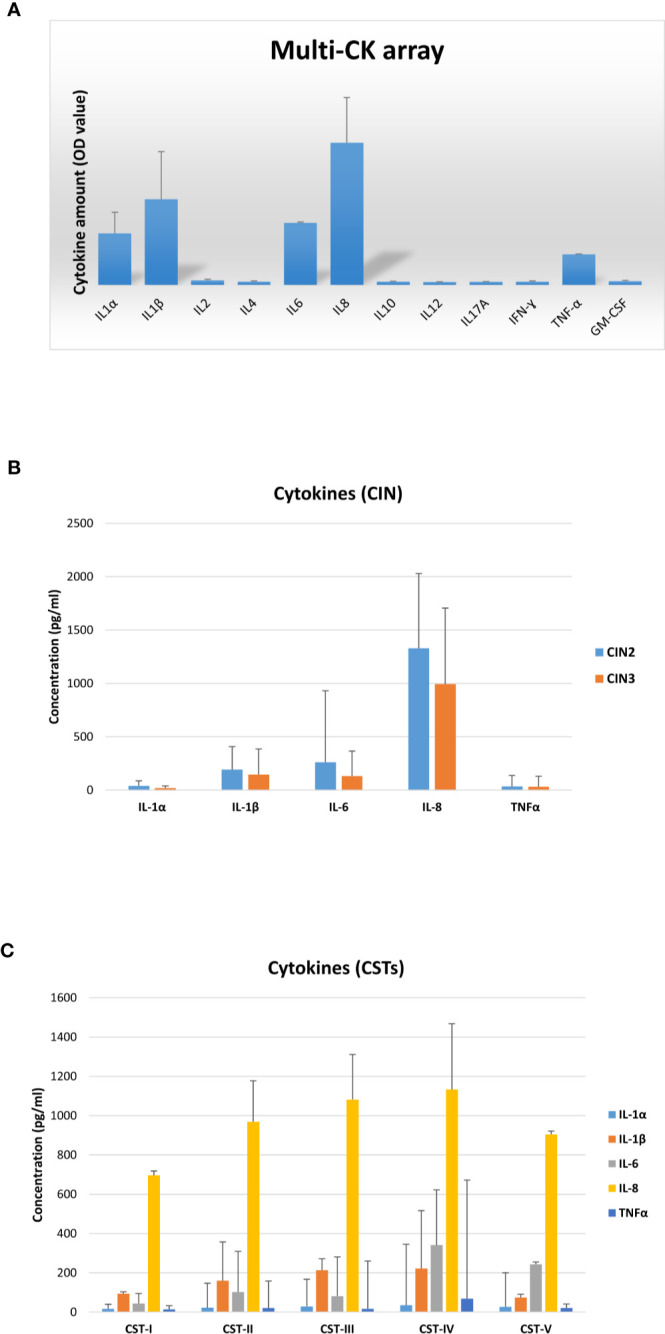
Cytokine profiles in vaginal washings from the CIN study cohort. **(A)** The results, obtained *via* a multi-analyte cytokine ELISA array performed on undiluted vaginal washings, are expressed as mean OD values ± SD (triplicate samples). **(B)** The results, obtained *via* individual cytokine ELISA assay for the indicated cytokines, performed on undiluted vaginal washing samples, are subdivided according to the lesion degree prior to intervention (CIN2 or CIN3) and expressed as mean pg/ml ± SD values of duplicate samples. **(C)** The results, obtained as described for panel B, are subdivided regarding the CST profiles of patients prior to intervention and expressed as mean pg/ml ± SD values of duplicate samples.

Each patient was also evaluated for the presence of cytokines/chemokines potentially associated with inflammation. First, vaginal samples were screened to detect 12 different cytokines/chemokines, and the results showed the presence of IL1α, IL1β, IL6, IL8, and TNFα (**Figure 9A**). Subsequent analysis of each cytokine by individual assay revealed fair amounts of each detected cytokine, with no statistically significant differences in cytokine concentration (mean pg/ml value) between the CIN2 and CIN3 subgroups (**Figure 9B**). In particular, the IL1α concentration corresponded to a mean value of 29.7 pg/ml (range 6.8–181.9 pg/ml), the IL1β mean concentration was 162.3 pg/ml (range 13.4–649.9 pg/ml), the IL6 mean concentration was 178.9 pg/ml (range 1.9–3493 pg/ml), IL8 presented a mean concentration of 1,105 pg/ml (range 284.9–2315 pg/ml), and the TNFα mean concentration was 33.4 pg/ml (range 6.3–633.3 pg/ml). Indeed, the cytokine concentrations differed accordingly with CST, particularly IL1β and IL8 in grouped CST-II, III and IV versus CST-I and V (*p* < 0.01), and IL6 and TNFα in the CST-IV profile versus the others (p < 0.05) (**Figure 9C**).

**Figure 10:**
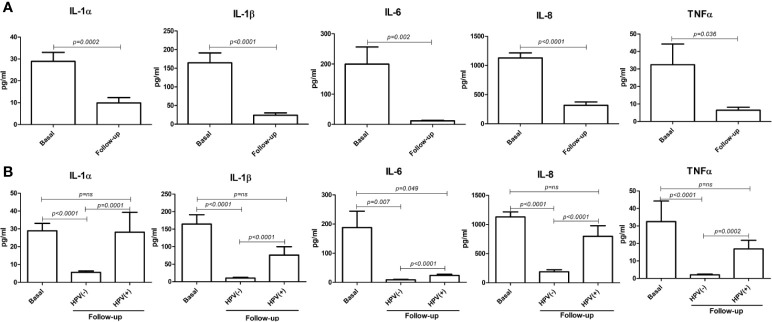
Comparison between basal and follow-up cytokine amounts in the vaginal washings of enrolled patients. All results were obtained by individual ELISA assays performed on undiluted vaginal washing samples and are expressed as mean pg/ml ± SD values of duplicate samples. **(A)** Comparison between the entire basal (72 patients) and follow-up (66 patients) groups. **(B)** Comparison between the basal group (72 patients) and the hrHPV-negative (52 patients) and hrHPV-positive (14 patients) subgroups at follow-up.

Interestingly, at the 6-month follow-up after LEEP, the concentrations of all detected cytokines were significantly decreased compared to their basal levels (*p* < 0.0001–0.05) (**Figure 10A**). However, the cytokine drop was almost exclusively significant in the hrHPV-cleared group, whereas no statistical significance (or no difference at all) could be detected between the basal and the follow-up levels of pro-inflammatory cytokines in the group of patients still showing persistence of hrHPV infection, with the exception of the IL-6 values, which were weakly significantly different in the hrHPV-persistent group compared to the original basal levels (p = 0.049) (**Figure 10B**).

## Discussion

Persistent infection with hrHPV is necessary but not sufficient for the development of CC, and recent studies focused on the possible role of the vaginal microbiome as a cofactor, due to its impact on the vaginal environment (Ferlay et al., 2018; Ferlay et al., 2019). Due to changess in the presence and metabolism of the microbial population of the vaginal tract, the vaginal environment could, in fact, facilitate or disadvantage infection by HPV and its stay in infected cells. However, longitudinal prospective studies characterising microbiota variations following CIN surgical removal are still needed, as well as functional studies on the compounds associated with specific vaginal microbiota profiles. Thus, in our study, we aimed to clarify the relationship between the vaginal microbiome, cytokine microenvironment, and hrHPV clearance/persistence in women surgically treated for CIN lesions, to highlight possible associations and provide new ideas and bases for CIN treatment.

The microbiome characterisation before the intervention showed, as expected, a significantly higher prevalence of the CST-IV (32.0%) profile compared to that among the healthy Caucasian population (10.3%) (Ravel et al., 2011), confirming the high prevalence of dysbiotic microbiomes in CIN women. Of note, CST-IV was significantly more prevalent in CIN3 compared to CIN2 patients, and CST-I was instead decreased, supporting a correlation of CST-IV with disease severity (OR=2.66, 95% CI 1.02–6.95, *p* < 0.01). Besides, the species identification/quantification provided by the used microarray technique highlighted the strict association between the absence of some specific *Lactobacillus* species and BV-associated microorganisms, evidencing the establishment of specific balances among microbes in the CIN habitat. Of note, *L. gasseri* dominance was associated with the absence of other Lactobacilli and presence of *C. albicans*, which was almost exclusively present in the CST-II microbiome. *L. iners* dominance was instead associated with high amounts of *U. parvum* and other BV-related bacteria.

Of note, the CIN2 microbiomes appeared to be still dominated by Lactobacilli, although accompanied by BV-associated bacteria and *C. albicans*, whereas CIN3 microbiomes showed a drop in Lactobacilli (except for *L. iners*) and high prevalence of *A. vaginae*, *G. vaginalis*, *U. parvum*, and *S. sanguinegens*, together with other species not frequent in CIN2 patients. Overall, the most frequently detectable species (present in more than 50% of samples) were, in order of frequency, *L. iners*, *G. vaginalis*, *F. magna*, *L. crispatus*, *P. asaccharolyticus*, *P. bivia*, *A. prevotii*, and *U. parvum.* These data are in line with previous reports showing a high frequency of vaginosis-associated microbes in HPV-infected women, accompanied by a decrease in Lactobacilli dominance (Kwasniewski et al., 2018; De Seta et al., 2019; Wakabayashi et al., 2019). No evidence of detectable amounts of *Chlamidia trachomatis* was found in our cohort, although it has been reported to be associated with CC induction (Karim et al., 2018). Similarly, the protozoon *Trichomonas vaginalis* was not evidenced in any sample from our study group, contrary to previous reports (Ghosh et al., 2017). However, significant differences have been reported in the prevalence of this microorganism relative to geographical and racial factors (Yang et al., 2018), and recent data showed anticancer activity of *Trichomonas* against CC cells, thus posing doubts about its role as an hrHPV cofactor (Zhu et al., 2018).

Overall, consistent with the presence of BV-associated microbiomes, a high concentration of pro-inflammatory cytokines was detected in the vaginal environment of CIN patients, including IL1α, IL1β, IL6, IL8, and TNFα, with the highest values found in the CST-IV profile compared to the others, confirming that BV-like vaginal microbiomes are associated with increased local inflammation (Campisciano et al., 2018; De Seta et al., 2019; Torcia, 2019).

Notably, a totally different picture was observed after surgical treatment, when a clear increase of CST-I/CST-III types, drop of CST-IV, and net augmentation of *L. crispatus* over the other species were observed, supporting its protective role in hrHPV eradication and consequent positive clinical outcomes. Importantly, such variations were exclusively present in the hrHPV-cleared group of patients (*p* < 0.001), whereas no significant changes were detected in the hrHPV-persistent group. In particular, an unmodified percentage of the subgroup still harboring hrHPV infection at follow-up presented the CST-IV profile (32 vs. 29%) (*p* = ns), although an increase of the CST-I type was also detectable in this subgroup. A constant trait of hrHPV-persistence-associated microbiomes was, however, the presence of *G. vaginalis*, which might likely initiate the polymicrobial biofilm present in bacterial vaginosis, allowing other species to adhere, including *A. vaginae*, as recently reported (Castro et al., 2020). No correlation was observed concerning the percentage of hrHPV clearance and the basal CST profile, but the very low number of patients in some CSTs rendered the comparison very difficult.

Concerning the risk of lesion recurrence, our data show that all recurrent patients were hrHPV-positive and 3 of them presented involvement of margins on excisional treatment, suggesting that although margin involvement is a risk factor for lesion recurrence, the post-treatment hrHPV-persistence predicts treatment failure more accurately than margin status and thus that post-treatment hrHPV testing is a more sensitive predictor of treatment outcome than margin involvement (Arbyn et al., 2017).

Interestingly, our data highlight the importance of the amount of microorganisms over their mere presence. In fact, when *L. crispatus*, although in a CST-I context, was accompanied by abundant *G. vaginalis*, *U. parvum*, and *C. albicans*, it was not *per se* favouring hrHPV clearance, as observed in the four patients developing CIN2/CIN3 clinical recurrence at follow-up. Consistent with this, such patients did not present any evident change of the microbiome profile at follow-up, except for a slight decrease of *L. crispatus* and increase of *L. gasseri/iners/jensenii* and *S. agalactiae*. Interestingly, this subgroup showed instead an increase in species mostly associated with aerobic vaginitis (AV), rather than BV, including aerobic enteric species such as *Streptococcus agalactiae* and group B Streptococci (Kaambo et al., 2018). Such a condition is characterized by a decrease in Lactobacilli and severe inflammation, and, although the number of recurrent patients was very low in the present study, this observation might deserve future studies to assess the association of such species with hrHPV persistence and related manifestations. In this direction, the increase of *L. gasseri*, *iners*, and *jensenii* in this subgroup is also of note, as no well-identified role has yet been recognized for them (Forney et al., 2006; Borges et al., 2014). However, given the small number of recurrences in our study, it is not possible to establish with certainty that some profile predisposes one to infection persistence or to lower success of surgical treatment, increasing the risk of recurrence. These data would need to be further investigated and confirmed in larger studies.

On the other hand, recent metabolome studies showed that a dysbiotic vaginal microbiome has a metabolic fingerprint that can discriminate patients from healthy subjects, and that non-*Lactobacillus* dominant profiles possess specific metabolic features (Ilhan et al., 2019; Borgogna et al., 2020). Considering the importance of host-microbe interactions in determining CC, and the possible translational potential of such data, it would therefore be important to study the correlations between specific vaginal microorganisms and metabolic hallmarks.

In conclusion, in our patient cohort, surgical removal of hrHPV-associated CIN lesions *per se* induced microbiome remodulation, and, on the other hand, specific microbiomes were associated with increased rates of hrHPV clearance.

Further studies enlarging the study population are needed to confirm our data; however, collected results suggest that interventions addressing vaginal microbiome modulation, through specific probiotics administration or by using targeted antimicrobials (antibiotics, bacteriophages), might be considered as potential tools to increase the rate of positive outcomes following surgical treatment of CIN lesions, opening the way to future possible therapeutic interventions.

## Data Availability Statement

The datasets generated for this study can be found in the BioStudies (https://www.ebi.ac.uk/biostudies/), accession No. S-BSST512.

## Ethics Statement

The studies involving human participants were reviewed and approved by Comitato Etico Unico della provincia di Ferrara, Azienda Ospedaliero-Universitaria di Ferrara, Ferrara, Italy. The patients/participants provided their written informed consent to participate in this study.

## Author Contributions

EC contributed the conception and design of the study, acquisition, analysis, and interpretation of data, and drafting the manuscript. MD’A and IS performed experiments and collected data. ES contributed to the design of the study and sample collection. SC contributed to patient recruitment and sample collection. CC contributed to the conception and study design. SM contributed to data analysis and revising the manuscript. PG contributed study design, data analysis, and revising of the manuscript. GB contributed the conception and design of the study, data analysis and interpretation, and revising of the manuscript. All authors contributed to the article and approved the submitted version.

## Funding

The project was supported in part by FAR 2018 (EC and GB) and Barion Vito Foundation.

## Conflict of Interest

The authors declare that the research was conducted in the absence of any commercial or financial relationships that could be construed as a potential conflict of interest.
