# Supplementary material for: Vaginal Microbiota and Cytokine Microenvironment in HPV Clearance/Persistence in Women Surgically Treated for Cervical Intraepithelial Neoplasia: An Observational Prospective Study
Source: Front Cell Infect Microbiol. 2020 Nov 5;10:540900. doi: 10.3389/fcimb.2020.540900 (PMC7676899; doi:10.3389/fcimb.2020.540900)
Supplement: Supplementary file 1 [file Table_1.docx]

***Supplementary material***

**Table S1.** Frequency of detection of the detected species in the basal microbiome of the study group and sub-groups.

| **Species** | **Frequency (%)^§^** | | |
| --- | --- | --- | --- |
|  | **hrHPV(+)** | **CIN2** | **CIN3** |
| *Lactobacillus iners* | 76.71 | 75.61 | 75.00 |
| *Gardnerella vaginalis* | 65.75 | **63.41** | **75.00** |
| *Finegoldia magna* | **65.75** | **73.17** | **59.09** |
| *Peptoniphilus asaccharolyticus* | 58.90 | 58.54 | 61.36 |
| *Lactobacillus crispatus* | **58.90** | **70.73** | **47.73** |
| *Prevotella bivia* | 54.79 | 53.66 | 56.82 |
| *Ureaplasma parvum* | 50.60 | 56.10 | 50.00 |
| *Anaerococcus prevotii* | 52.05 | **39.02** | **61.36** |
| *Anaerococcus hydrogenalis* | 42.47 | 41.46 | 40.91 |
| *Atopobium vaginae* | **42.47** | **29.27** | **52.27** |
| *Peptostreptococcus anaerobius* | 38.36 | **46.34** | **31.82** |
| *Streptococcus anginosus* | 36.99 | 39.02 | 38.64 |
| *Lactobacillus jensenii* | 38.35616 | **48.78** | **27.27** |
| *Sneathia sanguinegens* | 35.61644 | 41.46 | 34.09 |
| *Str. thermophilus/salivarius* | 41.09589 | 34.15 | 40.91 |
| *Bacteroides ureolyticus* | 36.9863 | 36.59 | 36.36 |
| *Fusobacterium nucleatum* | 36.9863 | **29.27** | **43.18** |
| *Lactobacillus gasseri* | 32.87671 | 34.15 | 34.09 |
| *Staphylococcus epidermidis* | 32.87671 | **39.02** | **29.55** |
| *Streptococcus mitis* | 32.24658 | 34.15 | 31.82 |
| *Porphyromonas asaccharolytica* | 31.50685 | 26.83 | 36.36 |
| *Prevotella disiens* | 31.50685 | **21.95** | **38.64** |
| *Parvimonas micra* | **30.13699** | **19.51** | **38.64** |
| *Lactobacillus vaginalis* | 27.39726 | **36.59** | **20.45** |
| *Mobiluncus curtisii* | 24.65753 | 29.27 | 25.00 |
| *Varibaculum cambriense* | 27.39726 | 29.27 | 25.00 |
| *Prevotella buccalis* | **26.0274** | **9.76** | **38.64** |
| *Streptococcus agalactiae* | 24.65753 | 26.83 | 22.73 |
| *Actinomyces naeslundii* | 24.65753 | 24.39 | 22.73 |
| *Fusobacterium periodonticum* | 23.28767 | 26.83 | 18.18 |
| *Corynebacterium aurimucosum* | 20.54795 | 19.51 | 22.73 |
| *Pan Aspergillus/Candida* | 20.54795 | 24.39 | 18.18 |
| *Aerococcus christensenii* | 20.54795 | **12.20** | **27.27** |
| *Leptotrichia amnionii* | 20.54795 | **12.20** | **27.27** |
| *Actinomyces odontolyticus* | 19.17808 | 17.07 | 20.45 |
| *Prevotella melaninogenica* | 20.54795 | 17.07 | 20.45 |
| *Enterococcus faecalis* | 17.80822 | 21.95 | 13.64 |
| *Propionibacterium acnes* | **16.43836** | **26.83** | **9.09** |
| *Veillonella parvula* | 19.17808 | 14.63 | 20.45 |
| *Mycoplasma hominis* | **15.06849** | **9.76** | **20.45** |
| *Actinomyces urogenitalis* | 12.32877 | 9.76 | 15.91 |
| *Bifidobacterium longum* | 12.32877 | **7.32** | **18.18** |
| *Lactobacillus salivarius* | 12.32877 | **19.51** | **4.55** |
| *Mobiluncus mulieris* | 10.9589 | 9.76 | 13.64 |
| *Str. intermedius/constellatus* | 12.32877 | 12.20 | 11.36 |
| *Bifidobacterium bifidum* | 9.589041 | 9.76 | 9.09 |
| *Prevotella nigrescens* | 9.589041 | 9.76 | 9.09 |
| *Ureaplasma urealyticum* | **9.589041** | **2.44** | **15.91** |
| *Candida albicans* | 9.589041 | 7.32 | 9.09 |
| *Candida krusei* | **6.849315** | **14.63** | **0.00** |
| *Eikenella corrodens* | 8.219178 | 9.76 | 6.82 |
| *Treponema pallidum* | 8.219178 | 7.32 | 9.09 |
| *Prevotella intermedia* | 6.849315 | 7.32 | 6.82 |
| *Staphylococcus arlettae/ saprophyticus* | 8.2191 | 9.76 | 4.55 |
| *Bacteroides fragilis* | 6.849315 | 4.88 | 6.82 |
| *Capnocytophaga gingivalis* | 5.479452 | 7.32 | 4.55 |
| *Staphylococcus aureus* | 5.479452 | 4.88 | 6.82 |
| *Actinomyces israelii* | 5.479452 | 7.32 | 2.27 |
| *Aerococcus urinae* | 5.479452 | 2.44 | 6.82 |
| *Bifidobacterium scardovii* | 4.109589 | 4.88 | 4.55 |
| *Campylobacter rectus* | 4.109589 | 0.00 | 4.55 |
| *Capnocytophaga sputigena* | 4.109589 | 7.32 | 2.27 |
| *Mycoplasma genitalium* | **5.479452** | **2.44** | **6.82** |
| *Porphyromonas gingivalis* | 5.479452 | 4.88 | 4.55 |
| *Selenomonas noxia* | 4.109589 | 4.88 | 4.55 |
| *Tannerella forsythia* | 5.479452 | 4.88 | 4.55 |
| *Bifidobacterium breve* | 4.109589 | **0.00** | **6.82** |
| *Campylobacter showae* | 2.739726 | 4.88 | 2.27 |
| *Candida parapsilosis* | 2.739726 | 2.44 | 4.55 |
| *Capnocytophaga ochracea* | 2.739726 | 4.88 | 2.27 |
| *Haemophilus ducreyi* | **2.739726** | **7.32** | **0.00** |
| *Pseudomonas aeruginosa* | 4.109586 | 2.44 | 4.55 |
| *Treponema denticola* | 4.109586 | 2.44 | 4.55 |
| *Trichomonas vaginalis* | **2.739726** | **4.88** | 2.27 |
| *Acidaminococcus fermentans* | 2.739726 | 2.44 | 2.27 |
| *Chlamydia trachomatis* | 2.739726 | **0.00** | **4.55** |
| *Neisseria gonorrhoeae* | 2.739726 | 2.44 | 2.27 |
| *Treponema socranskii* | 2.739726 | **0.00** | **4.55** |
| *Campylobacter gracilis* | 1.369863 | 2.44 | 0.00 |
| *Dialister pneumosintes* | 1.369863 | 2.44 | 0.00 |
| *Klebsiella granulomatis* | 1.369863 | 0.00 | 2.27 |
| *Lactobacillus acidophilus* | 1.369863 | 2.44 | 0.00 |

**^§^**bold characters evidence data significantly different between groups.
